# Characterization of Fungal *nirK*-Containing Communities and N_2_O Emission From Fungal Denitrification in Arable Soils

**DOI:** 10.3389/fmicb.2019.00117

**Published:** 2019-02-04

**Authors:** Huifang Xu, Rong Sheng, Xiaoyi Xing, Wenzhao Zhang, Haijun Hou, Yi Liu, Hongling Qin, Chunlan Chen, Wenxue Wei

**Affiliations:** ^1^Key Laboratory of Agro-Ecological Processes in Subtropical Regions and Taoyuan Station of Agro-Ecology Research, Institute of Subtropical Agriculture, Chinese Academy of Sciences, Changsha, China; ^2^College of Resource and Environment, University of Chinese Academy of Sciences, Beijing, China

**Keywords:** fungal denitrification, *nirK*, nitrous oxide, arable soil, soil type

## Abstract

Fungal denitrifiers play important roles in soil nitrogen cycling, but we have very limited knowledge about their distribution and functions in ecosystems. In this study, three types of arable soils were collected across different climate zones in China, including quaternary red clay soils, alluvial soils, and black soils. The composition and abundance of fungal *nirK*-containing denitrifiers was determined by MiSeq high-throughput sequencing and qPCR, respectively. Furthermore, a substrate-induced inhibition approach was used to explore N_2_O emissions from fungal denitrification. The results showed that the arable soils contained a wide range of *nirK*-containing fungal denitrifiers, with four orders and eight genera. Additionally, approximately 57.30% of operational taxonomic unit (OTUs) belonged to unclassified *nirK*-containing fungi. Hypocreales was the most predominant order, with approximately 40.51% of the total number of OTUs, followed by Sordariales, Eurotiales, and Mucorales. It was further indicated that 53% of fungal *nirK* OTUs were shared by the three types of soils (common), and this group of fungi comprised about 98% of the total relative abundance of the *nirK*-containing population, indicating that the distribution of fungal *nirK*-containing denitrifiers was quite homogenous among the soil types. These common OTUs were determined by multiple soil characteristics, while the composition of unique OTUs was manipulated by the specific properties of each soil type. Furthermore, fungal N_2_O emissions were significantly and positively correlated with fungal *nirK* abundance in the soils, whereas it was not clearly related to fungal *nirK* compositions. In conclusion, although the arable soils hosted diverse *nirK*-containing fungal denitrifiers, fungal *nirK* compositions were highly homogenous among the soil types, which could be a consequence of enduring agricultural practices. The abundance of fungal *nirK*-containing denitrifiers, rather than their composition, may play more significant roles in relation to N_2_O emission from fungal denitrification.

## Introduction

Nitrogen cycling processes are crucial for the sustainability of ecosystems (Zumft, 1997; Ishii et al., 2011); among them, denitrification is important in soil nitrogen transformation and can cause nitrogen loss. To date, denitrification has been widely investigated in various ecosystems (Philippot et al., 2007; Crenshaw et al., 2008). However, most studies targeted bacterial denitrifiers, and only few works involved fungal denitrifiers (Tiedje, 1994; Philippot et al., 2007; Mothapo et al., 2015). It was suggested that fungal denitrification may also play an important role in soil nitrogen cycling (Shoun et al., 1992; Maeda et al., 2015). According to previous studies, the contribution of fungi to N_2_O emission ranges from 17 to 89% in various soils (Laughlin and Stevens, 2002; McLain and Martens, 2006; Crenshaw et al., 2008). Cytochrome P450 (P450nor) was used as a molecular marker to target fungal denitrification since it is involved in the reduction of nitric oxide (NO) to N_2_O and is distinct from bacterial cytochrome *cb* type NoR (Shoun and Tanimoto, 1991; Shoun et al., 1992). However, since P450nor usually receives electrons from NADH to detoxify NO, it cannot act as a functional enzyme in the respiratory chain (Nakahara et al., 1993; Shoun et al., 2012). The Cu-containing nitrite reductase (NirK) enzyme catalyzes the reduction of ${NO}_{2}^{-}$ to NO (Nakanishi et al., 2010). Recently, some studies investigated fungal denitrification through *nirK*-containing communities due to DNA sequence data becoming available, allowing the use of PCR primers targeting the fungal *nirK* gene (Long et al., 2015; Wei et al., 2015). However, our understanding of the fundamental processes that underlie the distribution patterns of soil fungal denitrifiers remains limited, despite some studies suggesting that Hypocreales, Sordariales, and Eurotiales might be the dominant *nirK*-containing fungi in forest and arable soil samples (Long et al., 2015; Chen et al., 2016; Novinscak et al., 2016).

Compared with natural ecosystems, agricultural soils as a recent (decadal to centennial) anthropogenic ecosystem are characterized by intensive agricultural practices and have received high levels of fertilizers and regular soil management. Hence, the mechanisms shaping soil microbial community structures in agricultural soils may be more complex than those in natural ecosystems. Indeed, over the past decade, there has been growing evidence suggesting that management practices, such as land use change and human disturbance, could lead to significant variations in soil microbial communities at a given field site (Entry et al., 2008; Sheng et al., 2013). However, it has also been suggested that soil microbial communities may be mainly structured by soil properties, rather than agricultural practices (Thomson et al., 2015; Yao et al., 2017). Although we know little about the large-scale distribution patterns of functional microorganisms in agricultural soil ecosystems, some functional microbial groups, such as denitrifiers, behave differently compared to the overall microbial community in soils (Henry et al., 2006). It was reported that the community structures and functions of bacterial denitrifiers are more sensitive to anthropogenic influences (Chen et al., 2010; Liu et al., 2012; Gulden et al., 2015). Long-term fertilization and crop cultivation might enrich some bacterial denitrifying communities in agricultural soils. However, there is little knowledge regarding the distribution patterns and functions of fungal denitrifiers in agro-ecosystems.

The objective of this study was to investigate the composition, abundance and activity of fungal *nirK*-containing communities in different types of arable soils derived from various parent materials.

## Materials and Methods

### Sampling Sites

Samples from three types of arable soils were collected during July and August in 2014, including quaternary red clay soils (QRC), alluvial soils (AS), and black soils (BS). Ten sampling sites for each soil type were selected, and every two adjacent sampling sites were at least 10 km apart. The fields have history of cultivation with maize, wheat, soybean and oilseed rape, and the current crop was maize at the time of sampling. When the soil samples were collected from each site, a survey was conducted simultaneously. According to the survey, in recent years, N, P, and K fertilizers were generally applied annually at rates of between 105–135 kg N ha^-1^, 45–52 kg P ha^-1^, and 78–117 kg K ha^-1^. The sampling sites for QRC were located in Taoyuan (TY) and Qiyang (QY) of Hunan province in south China, with a mean annual temperature (MAT) and precipitation (MAP) of 23.25°C and 1349.00 mm, respectively. The sites for AS were located in Fengqiu (FQ) of Henan province and Luancheng (LC) of Hebei province in north China, with MAT and MAP of 13.35°C and 544.50 mm, respectively. The sites for BS were located in Gongzhuling (GZL) of Jilin province and Haerbin (HEB) of Heilongjiang province in northeast China, with MAT and MAP of 4.9°C and 574.00 mm, respectively. At each site, five soil columns (0–15 cm) were randomly taken with a soil sampler and well-mixed after removal of root residues. The sample was then divided into two portions: one of approximately 200 g was packed into a sterile plastic bag and transported to the laboratory on ice and stored at -80°C prior to molecular analysis. The remaining ∼1 kg was air-dried for assessment of soil physicochemical properties.

### Soil Physicochemical Properties

Total nitrogen (TN) was determined by Automatic Flow Injection after digestion in H_2_SO_4_. Total phosphorus (TP) and potassium (TK) were measure by wet digestion with sodium hydroxide and flame photometry, respectively. Soil organic carbon (SOC) was determined by K_2_Cr_2_O_7_ oxidation. Alkali-hydrolyzable nitrogen (AN) was measured by the alkali diffusion method (Bao, 2000). Available phosphorus (AP) was determined following extraction with 0.5 M NaHCO_3_, whilst atomic absorption spectroscopy (AAS) was used to determine available potassium (AK). Soil pH was determined at a soil-to-water ratio of 1:2.5. Soil moisture content was measured by comparison of fresh and dried (105°C; 24 h) weights of samples (Bao, 2000). Soil texture was measured using a fixed pipette method (Dane and Topp, 2002).

### DNA Extraction, PCR Amplification, and MiSeq Sequencing

Total soil DNA was extracted from 0.3 g of freeze-dried soil, according to Chen et al. (2010). The extracted DNA was quantified using NanoDrop ND-1000 spectrophotometer (Nanodrop Technologies, Wilmington, DE, United States). Fungal *nirK* fragments (ca. 480 bp) were amplified with a primer set targeting nirKfF/nirKfR (Wei et al., 2015). A two-step PCR was performed: for the first step, 25 μL PCR amplification solution contained 12.5 μL *Trans Tag* DNA polymerase High Fidelity Mix (TransGen Biotech), 3 μL DNA (105 ng), 1.5 μL of each primer (10 μmol L^-1^), and sterilized water. The reaction was initiated at 95°C for 3 min, followed by 10 cycles of 95°C for 45 s, 53°C for 30 s, 72°C for 50 s, with a final extension at 72°C for 5 min. Then, the PCR products were purified with an Agencourt AMpure XP kit (Beckman Coulter, Beverly, MA, United States) and eluted in 40 μL sterilized water. The second amplification step was carried out with 50 μL reaction solution containing 6 μL PCR product of the first step, 3 μL (10 μmol L^-1^) forward and reverse primers, 25 μL of *Trans Tag* DNA polymerase High Fidelity Mix and sterilized water to 50 μL. The amplifications were cycled for 25 cycles with the same program as the first round PCR. The PCR products were subjected to gel electrophoresis with 1.5% agarose. The bands of desired sizes were excised and purified using the Purelink Quick Gel Extraction kit (TransGen Biotech). The purified PCR products were sequenced by BGI Tech (Shenzhen, China) on an Illumina MiSeq platform.

### Quantification of Fungal and Bacterial *nirK* Genes

Fungal and bacterial *nirK* were quantified by qPCR targeting nirKfF/nirKfR (Wei et al., 2015) and 876F/1040R (Henry et al., 2004), respectively. The reaction mixture (10 μL) contained 5 μL SYBR green mix I, 0.2 μL Rox (Takara, Dalian, China), 0.2 μL (10 μmol L^-1^) of both forward and reverse primers, 1 μL (5 ng μL^-1^) of template DNA, and made up to 10 μL with deionized water. The thermal cycling program for fungal *nirK* was as follows: 95°C, 2 min; 40 cycles of 95°C for 15 s, 55°C for 30 s, 72°C for 30 s; 40°C for 30 s. The thermal cycling program for bacterial *nirK* was 95°C, 30 s; 40 cycles of 95°C for 5 s, 60°C for 30 s, 72°C for 10 s. Thermal programs were run with an ABI Prism 7900HT system (Applied Biosystems, Foster City, CA, United States) in triplicate. Standard curves for fungal and bacterial *nirK* genes were prepared using a 10-fold dilution series of a plasmid containing target gene fragments. To remove the contamination of humid acid in soil DNA, the qPCR data were corrected following the methodology of Wang et al. (2017).

### N_2_O Emissions Contributed by Fungi and Bacteria

To assess the contributions of fungi and bacteria to N_2_O emission, antibiotics cycloheximide, and streptomycin were used to inhibit fungal and bacterial protein synthesis, respectively. The procedures were carried out following Anderson and Domsch (1973). Briefly, based on antibiotic tests using cycloheximide and streptomycin over a series of concentrations in preliminary experiments, the minimum inhibitory concentrations of cycloheximide in QRC, BS, and AS soils were 8, 8, and 5 mg g^-1^, respectively, while that for streptomycin was 5 mg g^-1^ for all three types of soils. Soil samples (equivalent to 10 g dry weight) were put into a 125 mL amber jar followed by addition of 5 mL antibiotic solution, and was incubated at 4°C overnight. The jars were then transferred to a room at 27°C, and 5 mL of a solution containing 30 mg glucose and 1 mg KNO_3_ was added, followed by replacement of air with pure N_2_ three times. Then, 10% C_2_H_2_ was injected to inhibit the activity of N_2_O reductase, and the jars were incubated at 27°C for 30 h. Gas samples from the jars were measured every 6 h using a gas chromatograph equipped with an electron capture detector (Agilent 7890A). All treatments were set up in triplicate. Inhibitor additivity ratio (IAR) was used to determine whether cycloheximide and streptomycin exerted non-target effects (Beare et al., 1990). The time points when IAR = 1 were used for calculation of the proportions of N_2_O emission contributed by fungi or bacteria.

### Bioinformatic and Multivariate Statistical Analyses

Fungal *nirK* paired-end reads were demultiplexed in the different samples, according to exact matching to barcodes. Reads presenting one or more mismatches with the barcode sequences, or at least two mismatches with primer sequences, were discarded (Martin, 2011). The resulting reads were then merged using FLASH (Fast Length Adjustment of SHort reads), with a minimum of 10 bp in the overlapping regions (Magoc and Salzberg, 2011). Sequences exhibiting lengths outside the expected 200–500 bp range, or those containing any ambiguous bases, were removed. The consensus sequences of paired-end reads with a mean of 439 bp (after removing primers and adapters) were used for downstream analysis. Chimeras were removed using the Ribosomal Database Project (RDP) FunGene pipeline using default parameters (Fish et al., 2013). After filtering and chimera removal, *de novo* operational taxonomic unit (OTU) picking was performed using UCLUST at 97% sequence identity. Representative OTU sequences were translated into amino acid sequences using the FrameBot function of the RDP FunGene pipeline (Wang et al., 2013), and a local database containing 200 fungal *nirK* gene sequences downloaded from GenBank^1^ were used as reference templates. After further filtering singletons and OTUs detected in less than three soil samples, the retrieved amino acid sequences were used to construct a phylogenetic tree with MEGA 5.0 software suite (Tamura et al., 2011). The phylogeny tree was prepared using the ITOL webserver (Letunic and Bork, 2007). To acquire taxonomic identifies of the OTUs, taxonomy was assigned using the QIIME pipeline and the BLAST method. Venn diagrams were used to observe the shared and specific OTUs amongst the different soil types using R software (R Foundation for Statistical Computing, Vienna, Austria). Differences in community structure between samples were visualized using the weighted UniFrac distance (Lozupone and Knight, 2005) and non-metric multi-dimensional scaling (NMDS). Distance-based linear modeling was built using forward selection and 499 permutations of the data (McArdle and Anderson, 2001). Forward selection was used to determine the contribution of each variables in explaining the variations of fungal *nirK* community structure data (Anderson, 2003). Distance-Based Redundancy analysis (db-RDA), based on Bray–Curtis distances, was used to analyze environmental variables for the ordination of fungal *nirK* composition using Canoco 5.0 (Microcomputer Power, Ithaca, NY, United States) (Legendre and Anderson, 1999; ter Braak and Smilauer, 2012). Before conducted db-RDA analysis, the environment factors attributes were selected based on the variation inflation factors (VIFs), which stepwise removed redundant attributes, resulting in VIFs of less than 10 (O’Brien, 2007). Considering soil pH was widely recognized as a major driving factor in shaping soil microbial community structure in continental ecosystems (Lauber et al., 2009), it was included in the db-RDA despite its VIF > 10. Principal component analysis (PCA) was used to display distances between sites based on their soil attributes (environmental factors) across soil types.

One-way analysis of variance (ANOVA) was conducted to test for significant differences in soil physicochemical properties, the contributions of fungi or bacteria to N_2_O emission, and the abundance of fungal and bacterial *nirK* genes among the three soil types using SPSS software (version 18.0, Chicago, IL, United States). Pearson correlation analysis was performed to assess correlations between fungal and bacterial *nirK* gene copy number, and the contribution of N_2_O by fungi and bacteria, correlations between the ratios of fungal/bacterial abundance, and the ratios of fungal/bacterial contributions to N_2_O emission based on the log transformed data using SPSS software. Pearson correlation analysis involving fungal *nirK* abundance and environmental factors was also conducted with SPSS software.

### Accession Numbers of Nucleotide Sequences

All fungal *nirK* gene sequences obtained in this study have been deposited in the GenBank Sequence Read Archive (SRA) under the Accession No. SRP134068.

## Results

### Soil Physicochemical Properties

Soil physicochemical properties obviously differed among the three soil types (Table 1). Soil pH varied widely across soil types, while AS soils possessed the highest pH (8.27 ± 0.34) followed by BS (5.79 ± 0.42), QRC soils displayed the lowest pH (5.24 ± 0.65). In terms of soil texture, QRC soils contained the highest clay and lowest sand fractions (*P* < 0.01), while BS had slightly higher clay and lower sand contents than AS. For soil nutrient concentrations, BS soils possessed significantly higher SOC than QRC and AS (*P* < 0.05), whereas QRC and BS soils contained significantly higher levels of TN and TK than those in AS soils (*P* < 0.01). AS contained the highest TP with 0.85 g kg^-1^, followed by QRC, and BS possessed the lowest TP (*P* < 0.01). Furthermore, AS soils contained the lowest AN, and BS and AS soils possessed the highest AP and AK, respectively (*P* < 0.01).

**Table 1:** Soil physicochemical properties.

	TN^##^ (g kg^-1^)	TP (g kg^-1^)	TK (g kg^-1^)	SOC (g kg^-1^)	AN (mg kg^-1^)	AP (mg kg^-1^)	AK (mg kg^-1^)	pH	Sand (%)	Silt (%)	Clay (%)	Moisture content (%)
QRC^∗^	1.99 ± 0.14^#^ a^❈^	0.66 ± 0.20 b	22.27 ± 7.79 ab	13.86 ± 1.42 b	187.14 ± 17.99 a	14.70 ± 6.55 b	116.41 ± 54.63 c	5.24 ± 0.65 c	7.26 ± 4.02 b	53.60 ± 5.00 b	39.14 ± 3.44 a	25.08 ± 5.30 a
AS	1.39 ± 0.38 b	0.85 ± 0.04 a	18.45 ± 1.32 b	12.11 ± 2.79 b	136.42 ± 38.30 b	21.54 ± 13.10 b	248.74 ± 80.59 a	8.27 ± 0.34 a	15.46 ± 6.98 a	63.84 ± 3.57 a	20.70 ± 4.90 c	12.52 ± 6.01 b
BS	1.74 ± 0.36 a	0.33 ± 0.06 c	23.77 ± 4.53 a	16.51 ± 3.41 a	187.98 ± 33.57 a	42.75 ± 13.52 a	178.91 ± 47.01 b	5.79 ± 0.42 b	13.18 ± 2.11 a	62.69 ± 2.67 a	24.13 ±1.95 b	15.77 ± 5.47 b


*^∗^Soil types: AS, alluvial soils; BS, black soils; QRC, quaternary red clay soils.*

*^##^Soil physicochemical properties: TN, total nitrogen; TP, total phosphorus; TK, total potassium; AN, available nitrogen; AP, available phosphorus; AK, available potassium; SOC, soil organic carbon.*

*^#^Average ± standard deviation (*n* = 10).*

*^❈^Different letters (a, b, c) represent significant differences at *P* < 0.05.*

### Distribution Pattern of Fungal *nirK*-Containing Denitrifiers

**FIGURE 1:**
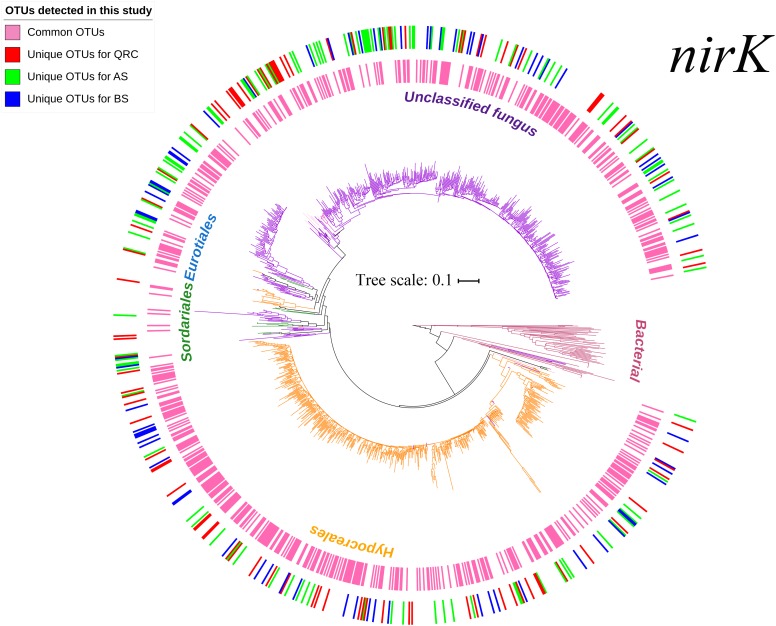
Neighbor-joining tree based on fungal *nirK* amino-acid sequences. Branches in different colors represent different fungal orders. Sequences originated from this study are highlighted with different colors in the circles, with the inner circles represent the common OTUs across the three soil types, and the outer circles represent the unique OTUs in different soil types. Different colors in the outer circles represent unique OTUs in different soils: Red-unique OTUs in QRC; Green-unique OTUs in AS; Blue-unique OTUs in BS. AS, alluvial soils; BS, black soils; QRC, quaternary red clay soils.

In total, 1,622,131 reads were generated by Miseq high-throughput sequencing, with an average length of 439 bp after removal of low-quality reads. After chimera checking, the remaining 1,257,313 sequences were grouped into 20,807 OTUs at 97% nucleotide sequence identity threshold. After FrameBot conversion, 9,146 OTUs with identity less than 50% of the reference sequences were filtered. Then, singletons and OTUs that occurred in less than three soil samples were also removed. The 1007 retrieved OTUs were finally used to construct a phylogenetic tree and for downstream analysis. According to the phylogenetic tree (Figure 1) and taxonomic distribution in Supplementary Figure S1A, the OTUs in the three types of arable soils were widely distributed across fungal taxonomies. Besides the unclassified taxa, *nirK*-containing fungi were manly affiliated to the four orders Hypocreales, Sordariales, Eurotiales and Mucorales, and eight genera *Fusarium*, *Trichocladium*, *Chaetomium*, *Talaromyces*, *Trichoderma, Aspergillus*, *Byssochlamys*, and *Actinomucor*. Among them, the most abundant order was Hypocreales, which comprised 40.51% of the total number of OTUs, and the most abundant genus was *Fusarium*, which comprised 40.22% of all OTUs. The remaining orders and genera represented minimal proportions. Two-dimensional NMDS visualization of the OTUs (Supplementary Figure S2) showed that there were no clear separations between the soil types, indicating that the community structures of *nirK*-containing fungi were quite similar in the different types of soils.

**FIGURE 2:**
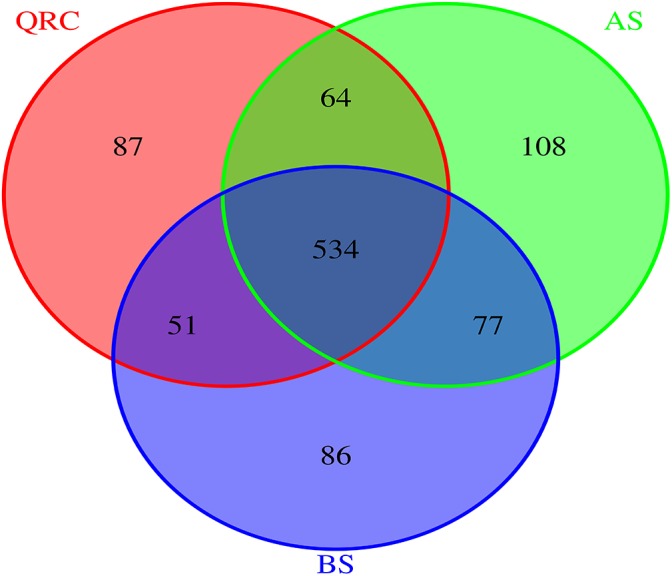
Venn diagram reveals the overlap of fungal *nirK* OTUs among three soil types. AS, alluvial soils; BS, black soils; QRC, quaternary red clay soils.

To better understand the distribution patterns of *nirK*-containing fungi in different types of soils, the communities were divided into three categories of “common,” “partially shared” and “unique” groups, based on the appearance of the OTUs among the soils. The OTUs that appeared in all of the types of soils were defined as common communities, OTUs appearing in any two types of soils were assigned as partially shared communities, and OTUs which only occurred in one type of soil were defined as unique communities. The results indicated that 534 of 1007 OTUs were classified as common, which accounted for approximately 53% of the total number of OTUs detected across the three types of soils (Figure 2). These common OTUs represented some 72.55, 68.20, and 71.39% of the total OTU numbers in QRC, AS and BS soils, respectively (Supplementary Table S1). In contrast, the unique OTUs only represented approximately 11.82, 13.79, and 11.50%, respectively.

These distribution patterns of fungal *nirK*-containing communities could be further identified by the relative abundance of different groups (Table 2). The data suggested that the common OTUs represented overwhelming proportions of the abundance of fungal *nirK* sequences in each type of soil, which were 98.09, 97.65, and 98.04% in QRC, AS, and BS soils, respectively. In contrast, the relative abundance of unique and partially shared OTUs only represented less than 1% of the overall abundance. Furthermore, it is interesting that the common OTUs covered six genera of *Chaetomium*, *Aspergillus*, *Talaromyces*, *Trichocladium*, *Trichoderma* and *Fusarium*, while those belong to *Actinomucor* and *Byssochlamys* only occurred as either unique or partially shared OTUs (Supplementary Figures S1B,C).

**Table 2:** Relative abundance of fungi *nirK* OTUs in different groups.

Group	Sub-group	QRC^∗^ (%)	AS (%)	BS (%)
Common OTUs	Common	98.09^❈❈^	97.65	98.04
Partial shared	QRC/AS	0.75	0.72	
	QRC/BS	0.51		0.48
	AS/BS		0.85	0.86
Unique OTUs	QRC	0.64		
	AS		0.77	
	BS			0.62


*^∗^Soil types: AS, alluvial soils; BS, black soils; QRC, quaternary red clay soils.*

*^❈^The relative abundance of common, partial shared, and unique groups to the whole population of fungal *nirK*-containing denitrifiers in each type of soils.*

### Variations in Fungal and Bacterial *nirK* Abundance, N_2_O Emissions From Fungal and Bacterial Denitrification

**FIGURE 3:**
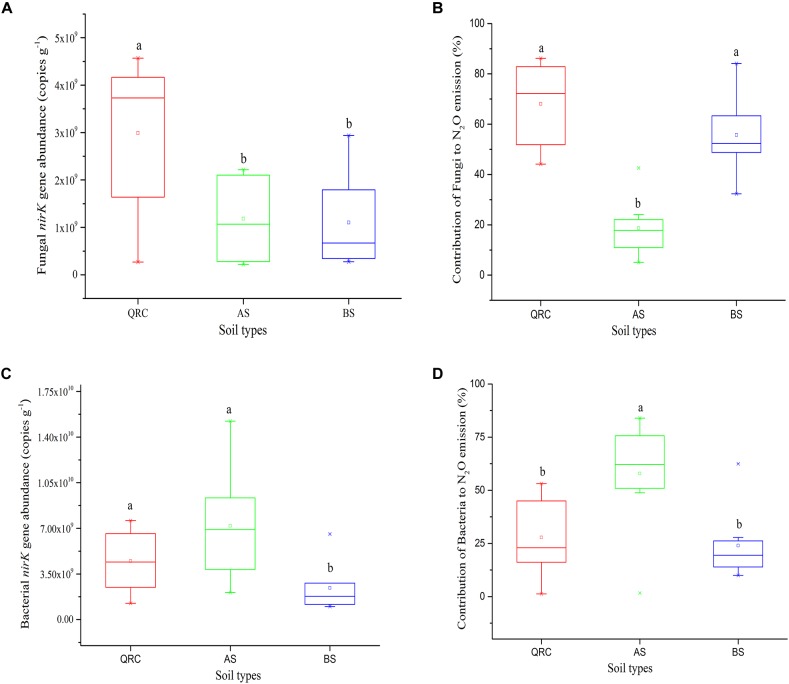
*nirK* abundance of fungal **(A)** and bacterial **(C)**, N_2_O emission from fungal **(B)** and bacterial **(D)** denitrification in three soil types. Different letters (a, b) represent significant differences among three soil types at *P* < 0.05 (*n* = 10). AS, alluvial soils; BS, black soils; QRC, quaternary red clay soils.

The abundance of the fungal *nirK* gene showed clear variations among the soil types (Figure 3A). The QRC soils contained the highest fungal *nirK*-type denitrifiers, with 3.15 × 10^9^ copies g^-1^ of soil, followed by the BS soils with 1.17 × 10^9^ copies g^-1^ of soil (*P* < 0.01), while the abundance of fungal *nirK*-containing denitrifiers in AS and BS exhibited no significant difference (*P* > 0.05). AS presented a lower abundance of about 9.92 × 10^8^ copies g^-1^ compared to the BS.

**FIGURE 4:**
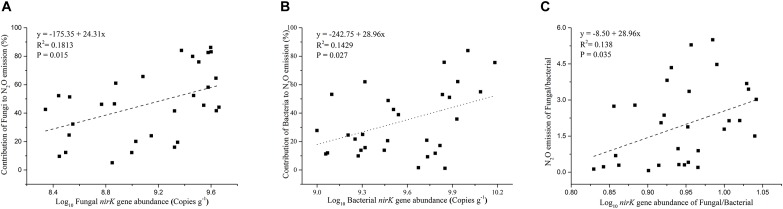
Relationship between soil fungal *nirK* gene abundance and the contribution of fungi to N_2_O emission **(A)**, soil bacterial *nirK* gene abundance and the contribution of bacteria to N_2_O emission **(B)**, ratios of fungal/bacterial *nirK* abundance and the ratios of fungal/bacterial contributions to N_2_O emission **(C)**.

The contribution of N_2_O emission caused by fungal denitrifiers was also determined in this study (Figure 3B). The QRC soils showed the highest N_2_O emission by fungal denitrification with 66.17%, followed by the BS soils with 53.34%. However, there was no significant difference between QRC and BS (*P* > 0.05). The lowest, with approximately 18.62%, occurred in the AS soils, which was significantly different to QRC and BS soils (*P* < 0.01). The scatter diagram clearly revealed that the abundance of the fungal *nirK* gene and N_2_O emissions by fungal denitrification exhibited a positive correlation (*P* < 0.05) (Figure 4A), which indicated that the population size of fungal *nirK*-denitrifiers may play important roles in the process of N_2_O production by fungal denitrification in arable soils.

The abundance of the bacterial *nirK* gene also differed significantly among the three soil types, with the AS soils possessing significantly higher abundance than QRC and BS soils (Figure 3C). The AS soils showed the highest N_2_O emission by bacterial denitrification, with 55.36%, followed by the QRC and BS soils with 27.44 and 21.66%, respectively, but there was no significant difference between QRC and BS (*P* > 0.05) (Figure 3D). The bacterial *nirK* gene abundance was also significantly and positively correlated with the bacterial contribution to N_2_O emissions (*P* < 0.05) (Figure 4B) but negatively correlated with the fungal contribution to N_2_O emission (*P* < 0.05) (Supplementary Figure S3). In addition, the ratios of fungal/bacterial *nirK* gene abundance were significantly and positively correlated to the ratios of fungal/bacterial contributions to N_2_O emissions (*P* < 0.05) (Figure 4C).

### Relationships Between Fungal *nirK*-Containing Communities and Environmental Factors

**FIGURE 5:**
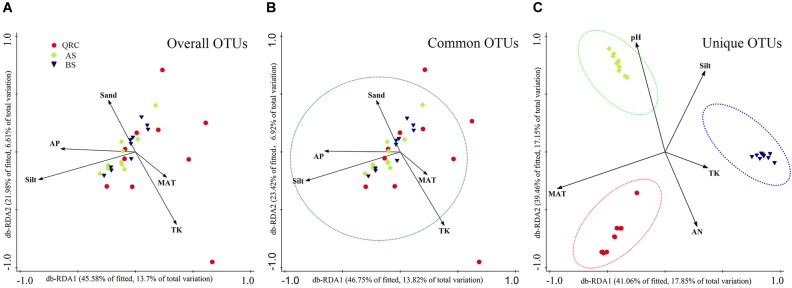
Distance based redundancy analysis (db-RDA) showing the relationships between overall **(A)**, common **(B),** and unique **(C)** fungal *nirK*-containing communities and environmental factors. db-RDA axes describe the percentage of the fitted or total variation explained by each axis while being constrained to account for group differences. Red circle- QRC, Green diamond- AS, Blue down triangle- BS. AS, alluvial soils; BS, black soils; QRC, quaternary red clay soils; TK, total potassium; AN, available nitrogen; AP, available phosphorus; MAT, mean annual temperature.

Distance-based RDA (db-RDA) analysis was used to reveal relationships between fungal *nirK*-containing community structures and independent environmental factors (Supplementary Table S2). Results showed that the overall composition of fungal *nirK*-containing communities was determined by multiple factors (Figure 5A). Based on the examined environmental factors, the cumulative variation of overall community structure explained by the first and second axes was 20.31%. Furthermore, these characteristics could explain approximately 20.74% of the variability in the distribution of common fungal *nirK*-containing denitrifiers (Figure 5B), and about 35% of the variations in the unique fungal *nirK*-containing denitrifiers (Figure 5C). Both for the overall and common communities, silt was the most significant factor in regulating the community structure (explaining 10.90 and 10.60% of the total variation, respectively), followed by TK (5.7 and 5.7% of the total variation, respectively) and MAT (5.5 and 5.2% of the total variation, respectively) (Supplementary Table S3). However, for the unique *nirK*-containing fungi, MAT (17.3%) and pH (16.7%) played more important roles in shaping the community structure. Pearson correlation analysis showed that the fungal *nirK* gene abundance was significantly and positively correlated with MAP, MAT, and clay content (*P* < 0.05), but negatively correlated to soil pH, AK, AP, sand, and silt contents (*P* < 0.05), indicating that fungal *nirK* abundance could be influenced by multiple environmental factors (Supplementary Table S4).

## Discussion

Fungal denitrifiers could be important microbes in soil nitrogen cycling in various ecosystems (Laughlin and Stevens, 2002; Herold et al., 2012; Wei et al., 2014), however, we have very limited knowledge regarding them. Since fungal *nirK* PCR primers were designed recently, investigation of fungal *nirK*-containing community compositions has been moved forward (Long et al., 2015; Maeda et al., 2015; Wei et al., 2015; Chen et al., 2016). In this study, a high diversity of *nirK*-containing fungal communities was detected in arable using high-throughput sequencing techniques. These included Hypocreales, Sordariales, Eurotiales, and Mucorales (Figure 1 and Supplementary Figure S1A). Nevertheless, more than half of the total number of OTUs were unclassified, which could further strengthen the diversity of *nirK*-containing fungi if they were classified. Among the assigned taxa, Hypocreales was predominant in the agricultural soils (Figure 1); its dominance has also been reported in forest, pastoral, and agricultural soils (Long et al., 2015; Wei et al., 2015; Novinscak et al., 2016; Xu et al., 2017). Considering that the coverage of the PCR primers used in this study was less than 100% (Chen et al., 2016), there might be other fungal *nirK*-containing denitrifiers that were not detected, and our understanding might be restricted to some degree.

Although we know a little about the distribution of fungal *nirK*-containing communities in environmental soils, wide variations are supposed to exist, based on the biogeographic distribution patterns of fungi (Tedersoo et al., 2014). It was identified that there were some variations of fungal *nirK*-containing communities among the sampling sites of each soil type from the NMDS (Supplementary Figure S2), especially for QRC soils. This scenario could be caused by variations in environmental factors, including soil physicochemical properties, land management practices, and climate conditions for each soil type. Since sampled AS and BS soils are located in the northern China plain, and in northeast China, respectively, where the cropping systems were relatively uniform and the soil formation process was relatively simple (Kendy et al., 2004; Kong et al., 2008), the consequent influences on soil properties were relatively small. QRC soils were distributed in hilly areas located in south China, where the land was intensively used for crop production by individual farmers, and differences in topography could also influence soil properties (He et al., 2007). Hence, variations in soil properties in QRC soils would be larger than in AS and BS soils. PCA, based on environmental factors, also suggested that the coefficient of variation of environmental factors was larger in QRC soils than that in AS and BS soils (Supplementary Figure S4), which could further support this speculation. Even with such variation, we found that fungal *nirK* composition was quite homogenous among the three types of soils because their common OTUs accounted for approximately 53% of the total number of OTUs, and represented over 98% of the relative abundance of fungal *nirK* populations (Figure 2 and Table 2). To understand these phenomena, related factors were taken into account. Because of the three types of soils being derived from different parent materials, their soil properties varied significantly between the soil types, including pH, SOC, and soil textures (Table 1). In addition, it was also observed that climatic conditions are obviously different between the locations of the three types of soil. Previous studies have suggested that soil properties are quite important in determining soil microbial community composition (Herold et al., 2014), and that climate factors could also severely influence soil microbial composition and structure (Tedersoo et al., 2014). Surprisingly, the community compositions of *nirK*-containing fungal denitrifiers were in a homogenous status, even with very different soil physicochemical conditions and climate factors between the soil types. These observations would strongly suggest that soil characteristics and climatic conditions would not be the key determinants in shaping soil fungal *nirK* composition in arable soils. Then the question is: which factors resulted in the distribution of fungal *nirK*-containing communities?

It is obvious that the common feature shared among these soils would be that they have been utilized in agricultural production for many years. Although there could be some dissimilarities in soil management by different farmers in various regions, such as crop rotation and fertilizer use, the majority of crops were basically similar, such as maize, wheat, soybean, and oilseed rape. The chronic accumulation of the effects of such agricultural practices could cause selective enrichment pressures on fungal *nirK* denitrifiers. As a consequence, the fungal *nirK*-containing communities exhibited a homogenous distribution in arable soils. Furthermore, taxonomic analysis also provided some clues on the effects of crop cultivation. Our results showed that most of the common *nirK*-containing denitrifying communities belonged to *Fusarium* (Supplementary Figure S1B), which is known to be widely distributed in cultivated soils, rather than in natural soils (Mothapo et al., 2013; Silvestro et al., 2013). Furthermore, it was reported that abundance of *Fusarium* was obviously increased with long-term fertilization (Liu et al., 2018), and most *Fusarium* spp. are linked to pathogens, which can be stimulated by continuous cropping (Bai et al., 2015).

It was also detected that the contributions of fungi to N_2_O emissions were clearly differentiated among the soils (Figure 3B). The QRC, BS, and AS contributed 66.17, 55.34, and 18.62%, respectively. N_2_O emission by fungal denitrification is either related to the denitrifying community abundance, or the community composition, especially those active components (Rutting et al., 2013; Xu et al., 2017). It has been documented that differences in soil properties can induce significant changes to the population size and community structure of soil denitrifiers (Henry et al., 2006). In particular, soil pH is recognized as an important factor in determining fungal population size by imposing a direct stress on fungal cells (Wheeler et al., 1991), and acidic soil is suggested to be suitable for fungal growth, whereas alkaline soil would restrict fungal development (Rousk et al., 2010). In the present study, we also found soil pH was significantly and negatively correlated to fungal *nirK* gene abundance (Supplementary Table S4), with the acidic QRC soils possessing the highest copy numbers of the fungal *nirK* gene, whereas, alkaline AS soils had the lowest abundance (Figure 3A). The results showed that fungal *nirK* abundance was positively correlated with clay content (Supplementary Table S4) and this was in agreement with previous studies showing that soils contained much clay can better retain water and nutrients and provide favorable conditions for microbial growth (Ciric et al., 2012). It is worth noting that beside edaphic factors, the climate conditions may also influence the abundance of soil fungi (Hawkes et al., 2011). In our case, we found a positive relationship among MAP, MAT, and soil fungal *nirK* abundance (Supplementary Table S4). Therefore, concerning the different types of soils distributed in different climate zones, the differences in fungal *nirK* abundance would be the consequence of interaction involving multiple factors, including soil physicochemical properties, and climate conditions, among others. However, the results also showed that fungal *nirK* abundance was negatively correlated with AK and AP contents (Supplementary Table S4). This finding conflicts with previous study which found fungal abundance to be positively correlated with nutrient status (Nottingham et al., 2017), especially in highly oligotrophic environments, nutrient limitation would be primary factors in determining fungal abundance (Arenz and Blanchette, 2011). In the present study, there were no limitations in nutrient availability in all soils (Zhou et al., 2016). Despite AS soils possessed higher AK and AP contents than QRC soils, the high pH would restrict the growth of soil fungi and result in lower fungal abundance than acidic QRC soils (Rousk et al., 2009), which led to above negative correlations.

Although the quantification of fungal *nirK* with the primer set of nirKfF/nirKfR would encompass some bacterial *nirK* genes because of the similarities between fungal and bacterial *nirK* genes, it was reported that the ratio of bacteria amplified by this primer set was around 7.8% (Chen et al., 2016). In addition, our results indicated that fungal *nirK* gene abundance was significantly and positively correlated with the fungal contribution to N_2_O emissions (Figure 4A), whereas bacterial *nirK* abundance was significantly and positively correlated to the bacterial contribution to N_2_O emissions (Figure 4B) but negatively correlated with fungal contributions to N_2_O emission (Supplementary Figure S3). Furthermore, the ratios of fungal/bacterial *nirK* gene abundance were also significantly and positively correlated to the ratios of fungal/bacterial contributions to N_2_O emissions (Figure 4C). These results would strongly suggest the abundance of fungal *nirK* can be used as a readily available indicator for fungal denitrification. It is worth noting that N_2_O formation by P450nor was also thought to occur exclusively in fungi, and the *p450nor* gene has been exploited as a distinctive biomarker in molecular assays to study fungal denitrifier diversity and abundance in the environment (Higgins et al., 2016; Novinscak et al., 2016). Recent studies based on genomic analysis revealed that only 48 of 167 *p450nor*-containing genomes harbored the *nirK* gene (Higgins et al., 2018), suggesting the *p450nor* is not always associated with the *nirK* gene. However, it was also reported that *p450nor* exhibits two- to five-fold more gene duplications than *nirK*, and *p450nor* is also involved in other functions, such as secondary metabolism, in addition to denitrification (Higgins et al., 2018). All of these results indicate a disconnect between *p450nor* presence and denitrification potential, hence the fungal *nirK* gene could be more closely linked to fungal denitrification.

## Conclusion

The arable soil hosted quite diverse *nirK*-containing fungal denitrifiers, involving four orders and eight genera. The predominant members of *nirK*-containing fungi belong to Hypocreales. A significant feature of fungal *nirK* distribution among the three types of arable soils was homogenization, which mainly resulted from the enrichment effects of agricultural practices, such as fertilization and cropping. N_2_O emissions driven by fungi were significantly and positively correlated to fungal *nirK* abundance, rather than composition of the soils.

## Author Contributions

HX, RS, and WW designed the experiments. WZ, HH, YL, HQ, and CC participated the study design and sample collection. HX, XX, and RS performed the experiments and analyzed the data. HX wrote the manuscript. WZ, RS, and WW edited the manuscript. All authors read and approved the final manuscript.

## Conflict of Interest Statement

The authors declare that the research was conducted in the absence of any commercial or financial relationships that could be construed as a potential conflict of interest.

## Footnotes

1 http://www.ncbi.nlm.nih.gov/genbank/

## Abbreviations

AS: alluvial soils
BS: black soils
QRC: quaternary red clay soils
