# Supplementary material for: Characterization of Fungal nirK-Containing Communities and N2O Emission From Fungal Denitrification in Arable Soils
Source: Front Microbiol. 2019 Feb 4;10:117. doi: 10.3389/fmicb.2019.00117 (PMC6369356; doi:10.3389/fmicb.2019.00117)
Supplement: Supplementary file 1 [file Data_Sheet_2.docx]

**SUPPLEMENTARY MATERIAL**


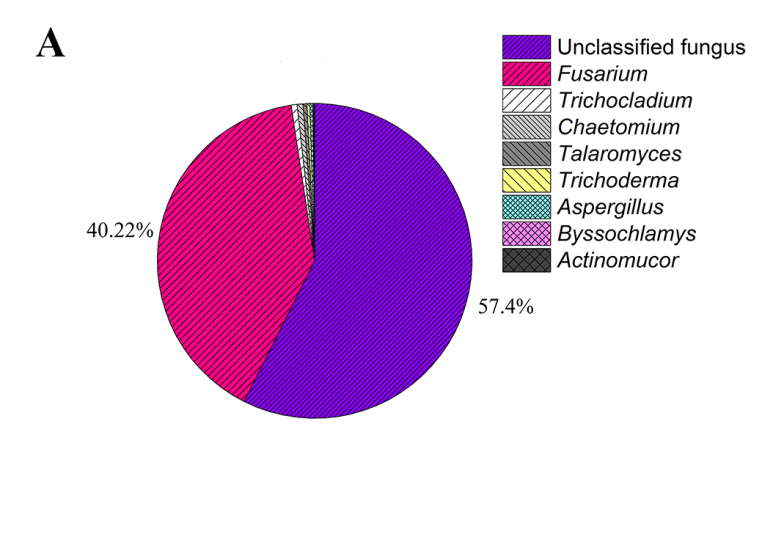
**
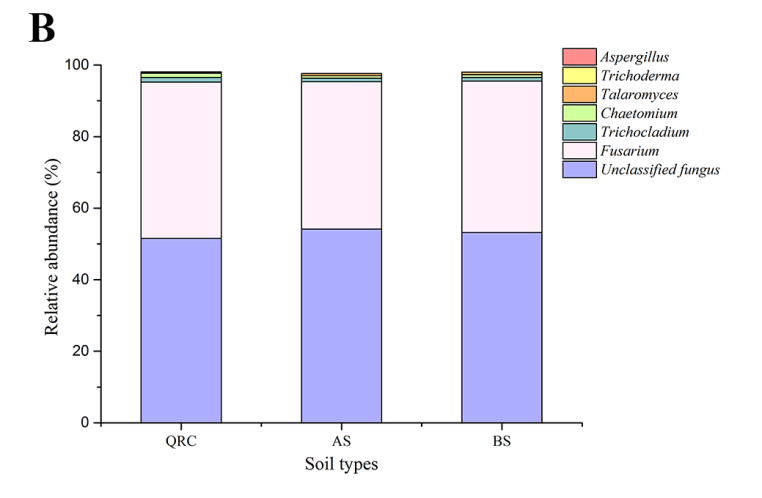

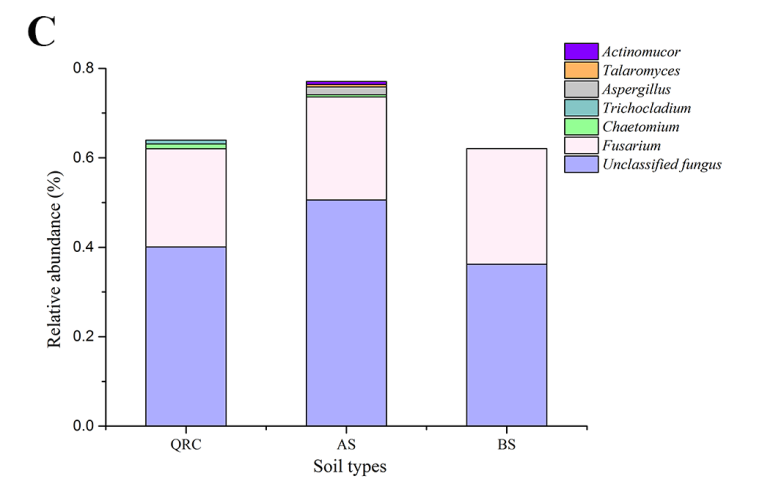
**

**FIGURE S1** Taxonomy analysis of overall **(A)**, common **(B)** and unique **(C)** fungal *nirK-*containing denitrifying communities at the genera level. The average relative abundance of genera in soil samples from each type of soils were shown in this figure (n=10) for QRC, AS and BS, respectively. QRC: quaternary red clay soils; AS, alluvial soils; BS, black soils.


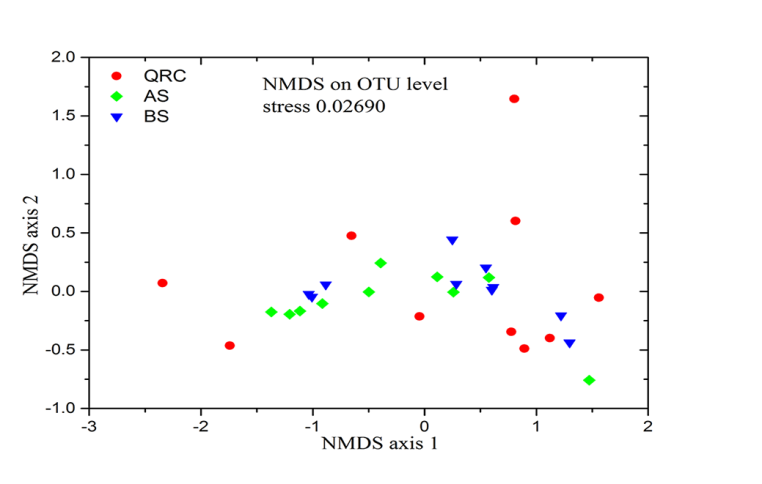


**FIGURE S2** Non-metric multidimensional scaling (NMDS) plot indicating the distance in fungal *nirK*-containing denitrifying community structure between different types of soils. The different color symbols represent fungal *nirK*-containing communities in different types of soils: Red circle-QRC, Green diamond-AS, Blue down triangle-BS. QRC: quaternary red clay soils; AS, alluvial soils; BS, black soils.





**FIGURE S3** Relationship between soil bacterial *nirK* gene abundance and the contribution of fungi to N_2_O emission.


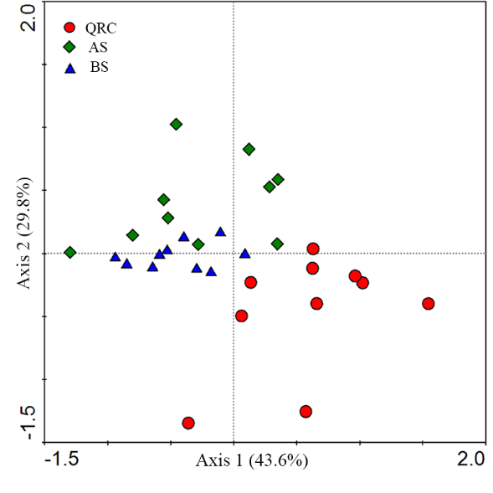


**FIGURE S4** Principal Component Analysis (PCA) based on environmental factors across soil types. QRC: quaternary red clay soils; AS, alluvial soils; BS, black soils.

**Table S1** Numbers and proportions of fungal *nirK* OTU in different group among three soil types.

|  | OTU numbers | | | Proportion (%) | | |
| --- | --- | --- | --- | --- | --- | --- |
|  | QRC^*^ | AS | BS | QRC | AS | BS |
| Overall | 736 | 783 | 748 | / | / | / |
| Common | 534 | 534 | 534 | 72.55 | 68.20 | 71.39 |
| Unique | 87 | 108 | 86 | 11.82 | 13.79 | 11.50 |

* Soil types: QRC: quaternary red clay soils; AS: alluvial soils; BS: black soils.

**Table S2** Variation inflation coefficient of environmental factors based on the community structure of overall, common and unique *nirK*-containing fungi.

|  | Overall | | Common | | Unique | |
| --- | --- | --- | --- | --- | --- | --- |
| Environmental factors^##^ | VIF^＊^ | VIF<10^#^ | VIF | VIF<10 | VIF | VIF<10 |
| TN | 7.76 | 7.17 | 7.75 | 7.16 | 8.37 | 7.67 |
| TP | 10.68 | 3.25 | 10.69 | 3.25 | 10.29 | 3.11 |
| TK | 5.05 | 2.02 | 5.05 | 2.02 | 5.05 | 1.86 |
| AN | 9.33 | 5.63 | 9.31 | 5.61 | 10.25 | 6.02 |
| AP | 4.10 | 2.86 | 4.10 | 2.86 | 3.95 | 2.77 |
| AK | 4.93 | 3.42 | 4.93 | 3.42 | 4.62 | 3.05 |
| pH | 21.39 | - | 21.32 | - | 25.28 | - |
| SOC | 3.07 | 2.32 | 3.07 | 2.32 | 3.18 | 2.18 |
| sand | 2.15× 10^6^ | 2.14 | 2.15× 10^6^ | 2.14 | 2.41× 10^6^ | 2.03 |
| silt | 2.26× 10^6^ | 2.90 | 2.26× 10^6^ | 2.90 | 2.37× 10^6^ | 2.81 |
| clay | 5.00× 10^6^ | - | 5.00× 10^6^ | - | 5.34× 10^6^ | - |
| MAT  MAP  Moisture content | 22.81  49.24  6.59 | 3.29  -  6.33 | 22.83  49.25  6.59 | 3.29  -  6.33 | 23.20  52.40  6.70 | 3.29  -  6.37 |

^##^ Environmental factors : TN: total nitrogen; TP: total phosphorus; TK: total potassium; AN: available nitrogen; AP: available phosphorus; AK: available potassium; SOC: soil organic carbon; MAT: mean annual temperature; MAP: mean annual precipitation.

^＊^VIFs before selection; ^#^ VIFs after selection (VIF<10).

**Table S3** The contributions of environmental factors to the variations in overall, common and unique fungal *nirK*-containing communities.

|  | Overall | | Common | | Unique | |
| --- | --- | --- | --- | --- | --- | --- |
| Environment factors^##^ | Explained (%) | *P* | Explained (%) | *P* | Explained (%) | *P* |
| Silt | 10.90 | 0.006 | 10.60 | 0.01 | 3.80 | 0.002 |
| TK | 5.70 | 0.064 | 5.70 | 0.076 | 3.00 | 0.086 |
| MAT | 5.50 | 0.038 | 5.20 | 0.062 | 17.30 | 0.002 |
| AP | 4.40 | 0.138 | 4.50 | 0.134 | 2.80 | 0.062 |
| Sand | 3.60 | 0.262 | 3.40 | 0.286 | - | - |
| AN | - | - | - | - | 2.60 | 0.22 |
| pH | - | - | - | - | 16.70 | 0.002 |
| Total | 30.1% |  | 29.4% |  | 43.4% |  |

^##^Environmental factors : TK: total potassium; AN: available nitrogen; AP: available phosphorus; MAT: mean annual temperature. The % represents the proportion that each environmental variable explained of the total variation.

**Table S4** Pearson’s correlation coefficients for relationship between fungal *nirK* abundance and environmental factors among three soil types

|  | TN^##^ | TP | TK | AN | AP | AK | pH | SOC | Sand | Silt | Clay | MAT | MAP | Moisture content |
| --- | --- | --- | --- | --- | --- | --- | --- | --- | --- | --- | --- | --- | --- | --- |
|  | （g kg^-1^） | （g kg^-1^） | （g kg^-1^） | (mg kg^-1^) | (mg kg^-1^) | (mg kg^-1^) |  | （g kg^-1^） | （%） | （%） | （%） |  |  | （%） |
| **Fungi *nirK* abundance** | 0.126 | -0.057 | 0.086 | 0.042 | -0.525** | -0.715** | -0.427* | -0.150 | -0.480* | -0.465* | 0.450* | 0.544** | 0.781** | 0.093 |

^##^ Environmental factors : TN: total nitrogen; TP: total phosphorus; TK: total potassium; AN: available nitrogen; AP: available phosphorus; AK: available potassium; SOC: soil organic carbon; MAT: mean annual temperature; MAP: mean annual precipitation.

*Correlation is significant at the 0.05 level；** Correlation is significant at the 0.01 level
